# Elucidating the relationships between host-associated (intestinal and gill) and environmental (water and sand) microbial communities across water exchange rates in *Babylonia areolata* aquaculture

**DOI:** 10.3389/fmicb.2026.1890193

**Published:** 2026-10-29

**Authors:** Di Tang, Zhigang Tu, Hongtao Liu, Feng Yu, Weiguang Zou, Minghui Shen

**Affiliations:** 1 Key Laboratory of Utilization and Conservation for Tropical Marine Bioresources of Ministry of Education, Hainan Tropical Ocean University, Sanya, China; 2 Hainan Provincial Key Laboratory of Tropical Maricultural Technologies, Hainan Academy of Ocean and Fisheries Sciences, Haikou, China; 3 College of Life Sciences, Hainan Normal University, Haikou, Hainan, China; 4 School of Marine Biology and Fisheries, Hainan University, Haikou, Hainan, China; 5 School of Marine Biology, Xiamen Ocean Vocational College, Xiamen, China

**Keywords:** 16S rRNA gene sequencing, bacterial biomarkers, ivory shell, SourceTracker, water quality

## Abstract

Water exchange is linked to survival in aquaculture, but its relationship with host–environment microbial interactions remains unclear. We aimed to characterize host-associated and environmental microbiota in *Babylonia areolata* (ivory shell) aquaculture, focusing on community relationships, environmental source contributions, associations with water quality, and host survival across water exchange rates. Ivory shells were reared for 60 days at high (100% d^−1^), medium (50% d^−1^), and low (0% d^−1^) water exchange rates. On day 60, water, sand, intestinal, and gill samples (12 samples per treatment) were profiled using 16S rRNA gene V3-V4 amplicon sequencing. The results showed that survival was lower only in the low-exchange group (*p* < 0.001). Analysis of similarities (ANOSIM) showed that the host microbiota differed significantly from environmental communities (R = 0.739, *p* = 0.001). SourceTracker identified sand as the largest environmental contributor; its contribution declined from 48.0 to 21.0% in the intestine and from 65.7 to 30.2% in the gills as the water exchange rate decreased. Reduced water exchange rates were also associated with declines in the relative abundance of intestinal *Nautella* and gill-associated *Ruegeria*. Linear discriminant analysis effect size (LEfSe) identified *Halodesulfovibrio* as a shared biomarker in both tissues under low water exchange conditions. Redundancy analysis (RDA) identified NO_3_^−^-N and pH as the environmental variables most strongly associated with environmental microbial community structure. Exploratory genus-level correlations showed that rearing water pH was negatively associated with *Halodesulfovibrio* in both tissues. Collectively, these results indicate that the water exchange rate is associated with host-associated microbial community composition, environmental source contributions, and ivory shell survival. Future studies should investigate the mechanisms underlying the associations among environmental microbial communities, water quality conditions, and host survival, thereby informing microecological management in low water exchange aquaculture systems.

## 1 Introduction

In aquaculture systems, microbial communities play key roles in ecological processes including material cycling and energy flow. Host-associated microbiota may modulate host physiological functions through immune and metabolic interactions (Zhang et al., 2019), while environmental microorganisms are associated with animal health by maintaining water quality and balancing ecosystem processes (Wang et al., 2021). Therefore, the roles of host and environmental microorganisms should be carefully considered when assessing and managing aquaculture systems.

The intestinal tract is crucial for nutrient absorption, with the intestinal microbiota being abundant, structurally complex, and functionally diverse. This microbiota is closely associated with the host, significantly influencing nutrient digestion, physiological metabolism, and disease resistance (Zhao et al., 2025; Talwar et al., 2018; Xiao et al., 2021). The gut microbiota of aquatic animals is influenced by intrinsic factors such as feeding habits, growth stages, and physiological states, as well as extrinsic factors such as habitat conditions and dietary strategies (Chen et al., 2022). Existing evidence suggests a bidirectional interaction between the culture environment and the gut microbiome, where the bacterial communities surrounding the host’s gut microbiota can influence its composition and structure. The colonization of the gut microbiota can, in turn, affect the microbial community in the surrounding culture environment, reflecting the host’s adaptability to the culture environment (Sullam et al., 2012; Zhang et al., 2019). The culture environment (pH, dissolved oxygen, temperature, and salinity) influences the composition of environmental microorganisms, ultimately affecting the intestinal microbiota (Giatsis et al., 2015; Tang et al., 2014; Zheng et al., 2021). When pathogenic bacteria in the environment and host exceed critical thresholds, disease outbreaks occur (Yao et al., 2018). While host–gut microbiota interactions are well documented (Adamovsky et al., 2018; Apprill, 2017; Strand et al., 2021), interactions between the gut microbiota and the environment in aquaculture systems remain poorly understood. Gills serve as a primary barrier in aquatic animals and the first line of defense against environmental elements entering the host (Peatman et al., 2015; Gomez et al., 2013). The gill surface is typically covered by a constantly renewed mucus layer containing immunologically active molecules derived from the underlying lymphoid tissue. These molecules, akin to immune molecules in the gut, help block opportunistic pathogens (Magnadottir, 2010; Peatman et al., 2015; Larsen et al., 2015). In addition, indigenous microorganisms, primarily bacteria, colonize the gills through spatial and nutritional competition, providing immune, protective, and regulatory functions essential for host adaptation and health (Balcázar et al., 2006). Recent studies have explored the antimicrobial role and environmental stress adaptation of gills (Hess et al., 2015); however, information on the relationship between the culture environment and the gill microbial community remains limited.

*Babylonia areolata*, commonly referred to as the ivory shell, is a member of the Babylonidae family. This species is widespread in the tropical and subtropical regions of the Indo-Pacific Ocean (Zou et al., 2024). As an economically important marine gastropod, it is considered a dominant aquaculture species due to its high value and rapid growth characteristics. However, as an invertebrate, the ivory shell lacks a specialized immune system to withstand environmental stressors and pathogens (Di et al., 2013), making it particularly vulnerable in high-density aquaculture settings compared to the wild. This vulnerability poses a serious threat to the stability and sustainability of its aquaculture production (Ruangsri et al., 2018; Zhou et al., 2023; Ding et al., 2024). Consequently, industry experts are exploring alternative culture techniques, such as closed aquaculture systems isolated from external environments. These systems offer benefits such as protection from diseases and pathogens, controlled growth conditions, and proximity to markets (Kuhn et al., 2013). However, our recent experiments revealed that closed systems without water exchange significantly reduced growth in ivory shells. Similar observations have been reported for fish species, including common carp (*Cyprinus carpio*) and Nile tilapia (*Oreochromis niloticus*), in which water exchange modulates metabolic waste accumulation that directly dictates growth performance (Martins et al., 2009a, 2009b). The mechanisms through which limited water exchange affects ivory shells remain unclear. Recent findings suggest that the aquaculture environment is intricately linked to the physiology, immunity, and growth of ivory shells. Notably, nitrite nitrogen correlates significantly with heterotrophic bacteria and *Vibrio* in the intestines (Hui et al., 2018), while factors such as nitrite nitrogen, pH, salinity, and temperature markedly influence antioxidant and immune enzyme activities in the intestines and gills (Lü et al., 2017; Liu et al., 2023; Ding et al., 2024). Despite the critical role of the intestinal and gill microbiomes as key interaction nodes with environmental microorganisms (Legrand et al., 2017; Read and Holmes, 2017), research in this area remains limited, and the comprehensive effects of the aquaculture environment on these microbial communities are not yet fully understood.

Given the substantial differences in habitat characteristics, community structure, and functional traits between waterborne and sand-dwelling microorganisms (Lokesh and Kiron, 2016; Zhou et al., 2022), the differential shifts in water and sand microbial communities driven by varied water exchange rates may crucially influence host microbiome formation and environmental adaptation. The primary objectives of the study were: (1) to characterize the microbial community structure in the intestine, gills, sand, and water of the ivory shell aquaculture system and assess the effects of water exchange on these microbial communities; (2) to quantify the relationships between the intestinal, gill, and environmental (sand and water) microorganisms at different water exchange rates; and (3) to analyze environmental factors affecting the host-associated microbial community and explore the aquaculture environment’s potential impact on pathogenic bacteria in the intestine and gills of ivory shells. This research aimed to elucidate the interactions between host-associated microbial communities and the aquaculture environment, thereby providing scientific support and a theoretical foundation for optimizing aquaculture conditions and preventing related diseases.

## 2 Materials and methods

### 2.1 Experimental design

This study was conducted at the Qukou breeding base of the Hainan Academy of Ocean and Fisheries Sciences (Haikou, China). All ivory shells were derived from a single parental cohort obtained from the same population, with larvae originating from synchronized spawning events to ensure developmental uniformity. Based on customary production practices and preliminary experiments, the traditional flow-through aquaculture of ivory shells typically employs a 100% daily water exchange rate. Reducing the water exchange rate may negatively affect both the aquaculture environment and the vitality of ivory shells. To investigate the influence of water exchange rates, particularly low rates, on the microbial characteristics of ivory shells and their relationships with the rearing environment, three water exchange rates were tested: High (100% d^−1^), medium (50% d^−1^), and low (0% daily water exchange; closed recirculation without water renewal). Each treatment comprised two independent 180-L tanks, each equipped with a recirculation pump and coral biofilter. In the high and medium water exchange treatments, each tank received flow-through seawater at rates of 7.5 and 3.75 L·h^−1^, corresponding to daily replacement of 100% (180 L) and 50% (90 L) of the tank volume, respectively. Tanks in the low water exchange treatment were operated as closed recirculating systems without water renewal.

The experiment involved ivory shell individuals with an average weight of 3.2 ± 0.2 g and a length of 23.3 ± 1.9 mm, which were acclimated for 15 days. A total of 900 healthy and vigorous ivory shell individuals were randomly distributed among 18 plastic cages (length × width × height = 20 cm × 15 cm × 24 cm, with 3 cm of sand at the bottom), with 50 individuals per cage. In total, three cages were placed in each tank, resulting in six cages per treatment. The water exchange rate was applied independently at the tank or water system level. Therefore, the tank/water system was treated as the experimental unit, whereas cages within each tank were treated as subsamples. Throughout the experiment, seawater parameters were maintained at stable levels, including sufficient dissolved oxygen, temperature (26.0 ± 1.0 °C), and salinity (33.0 ± 0.5‰). Ivory shells were fed a compound diet equivalent to 3.0% of their body weight daily. Leftover feed, feces, and dead individuals were removed promptly. The experimental design, sampling scheme, sample processing, and analytical workflows are summarized in Figure 1.

**Figure 1:**
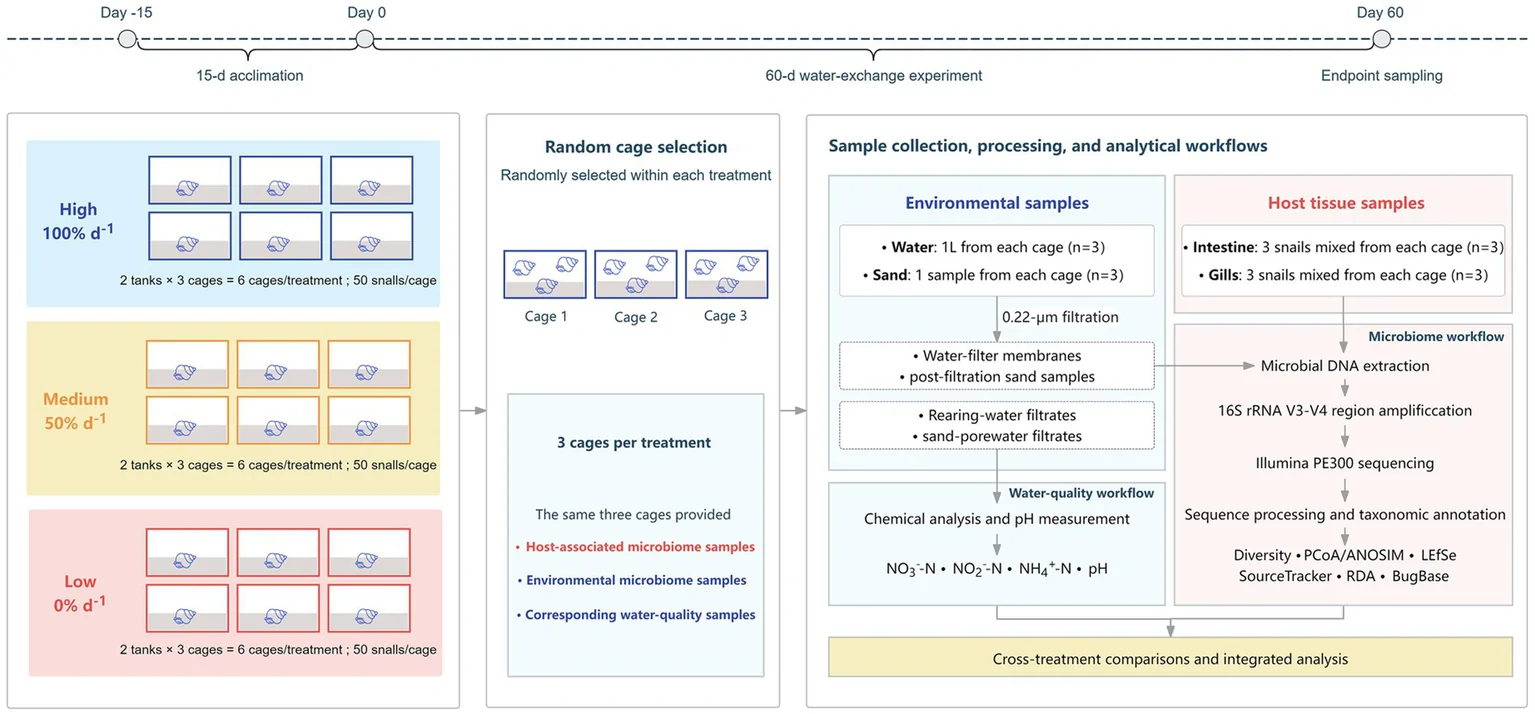
Experimental design, sampling scheme, and analytical workflows for *Babylonia areolata* under different water exchange conditions. Ivory shells were acclimated for 15 d and subsequently reared for 60 d under high (100% d^−1^), medium (50% d^−1^), or low (0% d^−1^) water exchange conditions. Each treatment comprised two independent tank/water systems, with three cages per tank. For microbiome profiling, all three cages within a single tank in each treatment were sampled for host tissue and environmental analyses (*n* = 1 tank per treatment). Intestinal and gill tissues from three snails within each sampled cage were mixed to generate one pooled sample per tissue. Water samples were filtered through 0.22-μm membranes, while sand porewater was obtained by vacuum filtration of sand samples through identical membranes. Water filter membranes and post-filtration sand samples were used for microbiome analysis, whereas the corresponding rearing water and sand porewater filtrates were analyzed for NO_2_^−^-N, NO_3_^−^-N, NH_4_^+^-N, and pH. Microbial communities were characterized using 16S rRNA gene sequencing, followed by cross-treatment comparisons and integrated analysis with water quality variables.

### 2.2 Sampling procedure

The experiment lasted for 60 days, and ivory shells were sampled 72 h after the final feeding. Surviving individuals in all 18 cages, with six cages per treatment, were counted to determine cage-level survival. For microbiome profiling, three cages were sampled from a single tank in each treatment (n = 1 tank per treatment). Samples collected from these cages therefore represented technical subsamples of the same tank environment rather than independent experimental units. Although included as separate observations in Analysis of similarities (ANOSIM), redundancy analysis (RDA), and envfit, the resulting statistics were interpreted descriptively and do not support replicated treatment-level inference. In total, three individuals were randomly selected from each sampled cage, yielding nine individuals per treatment. The external surfaces of the specimens were wiped with 75% ethanol. Subsequently, in an ultra-clean workbench, the intestines (I) and gills (G) of the ivory shells were removed using sterilized anatomical tools and placed in sterile 2-ml cryopreservation tubes, which were stored at −80 °C after cold shock in liquid nitrogen. Given the limited biomass of intestinal and gill tissues in ivory shells, three intestines (or three gills) from each cage were pooled into one sample. This procedure yielded three pooled cage-level subsamples per treatment for 16S rRNA gene sequencing. Although pooling precludes the estimation of individual-level variance and may reduce sensitivity to within-group variation, this approach was necessary to ensure adequate material for subsequent analysis.

Environmental samples were obtained from the aforementioned three cages. In total, three sand (S) subsamples were collected from each cage and combined in a sterile tube to form one composite sample weighing approximately 10 g, yielding three sand subsamples per treatment. Water (W) samples were collected from below the water surface in each of the same three cages, yielding three water subsamples per treatment. After collection, 1,000 mL of each water sample was vacuum-filtered through a 0.22-μm polycarbonate membrane (Biosharp, Anhui, China), and each membrane was placed in a sterile centrifuge tube. Sand porewater was obtained by vacuum filtration of each sand sample through an identical membrane. The sand samples and water-sample filter membranes were immediately frozen in liquid nitrogen and stored at −80 °C. The resulting rearing water and sand porewater filtrates were analyzed for nitrite nitrogen (NO_2_^−^-N), nitrate nitrogen (NO_3_^−^-N), and ammonia nitrogen (NH_4_^+^-N) using an automated discrete chemical analyzer (CleverChem 380, DeChem-Tech GmbH, Germany) according to China’s National Standard GB 17378.4–2007. Water temperature, salinity, and pH were measured using a portable multiparameter analyzer (SG78, Mettler-Toledo, Shanghai, China).

### 2.3 Deoxyribonucleic acid (DNA) extraction and polymerase chain reaction (PCR) amplification

Total microbial DNA was extracted from approximately 2–3 g of the water filter membrane, sand, gill, and intestinal samples, using the FastPure Soil DNA Isolation Kit (Magnetic Bead) (MJYH, Shanghai, China). The quality of the extracted genomic DNA was examined using 1% agarose gel electrophoresis, and DNA concentration and purity were determined using NanoDrop 2000 (Thermo Scientific, USA).

Primers 338F (5’-ACTCCTACGGGAGGCAGCAG-3′) and 806R (5’-GGACTACHVGGGTWTCTAAT-3′) (Liu et al., 2016) were used to amplify the V3-V4 hypervariable region of the 16S rRNA gene, producing amplicons of approximately 468 base pairs (bp). The polymerase chain reaction (PCR) program used was as follows: initial denaturation at 95°C for 3 min; 30 cycles of denaturation at 95°C for 30 s, annealing at 53°C for 30 s, and elongation at 72°C for 45 s, and final elongation at 72°C for 10 min. The PCR product was extracted from a 2% agarose gel, purified using the PCR Clean-Up Kit (YuHua, Shanghai, China) according to the manufacturer’s instructions, and quantified using a Qubit 4.0 (Thermo Fisher Scientific, USA). The purified PCR products were pooled in equimolar amounts and paired-end sequenced (2 × 300 bp) on an Illumina PE300 platform (Illumina, San Diego, USA) according to the standard protocols by Majorbio Bio-Pharm Technology Co., Ltd. (Shanghai, China). No extraction blanks were included during DNA extraction; however, PCR negative controls with sterile water were included to monitor potential contamination during amplification.

### 2.4 Data processing and statistical analysis

Cage-level survival counts from all 18 cages nested within six tanks were analyzed using a binomial generalized linear mixed model with a logit link in R 4.4.3 using the glmmTMB package. The numbers of surviving and dead individuals in each cage were specified as a grouped binomial response, with water exchange rate as a fixed effect and tank as a random intercept. To evaluate the necessity of the tank random effect and to check for overdispersion, we fitted an observation-level random effect (OLRE) model and a beta-binomial model. The overall treatment effect was assessed using a likelihood-ratio test, and pairwise contrasts were used to estimate odds ratios (ORs) and their 95% confidence intervals (CIs). All other R-based statistical analyses were performed using R version 3.3.1. Statistical significance was evaluated at α = 0.05. Raw FASTQ files were demultiplexed using an in-house Perl script, quality-filtered using fastp (v 0.23.4), and merged using FLASH (v 1.2.11). To eliminate sequencing-depth bias in α- and β-diversity analyses, all samples were rarefied to the depth of the smallest sample (39,961 clean reads), yielding an average Good’s coverage of 99.1%. Representative operational taxonomic unit (OTU) sequences were aligned against the SILVA database (v 138.2), and species classification was annotated using the RDP Classifier (v 11.5) with a confidence threshold of 0.7. QIIME (v 1.9.1) was used to calculate the Shannon and Chao1 diversity indices.

The α-diversity indices were compared using the Kruskal–Wallis *H* test at two grouping levels: (1) the four major sample types (I, G, S, and W) and (2) the 12 treatment groups (IH, IM, IL, GH, GM, GL, SH, SM, SL, WH, WM, and WL). False discovery rate (FDR) correction was applied separately to each family of tests to account for multiple comparisons, with statistical significance set at an FDR-adjusted p-value of < 0.05. For comparisons among the treatment groups, only significant differences within the same sample type were reported. Venn diagram analysis was performed using the VennDiagram package in R to identify shared and unique OTUs among groups. The biomarkers of each group were screened using the linear discriminant analysis effect size (LEfSe) software (v 1.0), where the threshold for linear discriminant analysis (LDA) was set to 4.0 with a p-value cut-off of 0.05. The contribution of environmental microorganisms to the host microbiome of ivory shells was quantified using the R SourceTracker package (Knights et al., 2011). PCoA was conducted using the Bray-Curtis distance with the Vegan package in R, and plots were generated using the ggplot2 package in R. RDA was performed using the vegan package (v 2.5.3) in R to clarify the impact of environmental factors on community composition. Bacterial microbiome phenotypes were classified using BugBase against the Greengenes database gg_13_5. Differences in function among groups were calculated using the Kruskal–Wallis *H* test with the Vegan package in R.

### 2.5 Ethics statement

All studies presented in this manuscript were conducted in accordance with the ethical standards of the Hainan Academy of Ocean and Fisheries Sciences and were approved by the Animal Care Committee of the Hainan Academy of Ocean and Fisheries Sciences (EAEC-HAOFS-No.2026001). As these field experiments were not conducted in a protected area and ivory shell is not an endangered or protected species, no additional permission was required.

## 3 Results

### 3.1 Sequencing depth and α-diversity

After 60 days, treatment-level survival rates were 98.7%, 96.7%, and 86.7% under high, medium, and low water exchange conditions, respectively. Tank-level survival was 98.7% in tanks H1 and H2, 97.3% in M1, 96.0% in M2, and 86.7% in L1 and L2 (Figure 2A). The binomial GLMM, with tank included as a random intercept, estimated a tank-level variance of 1.604 × 10^−10^ (SD = 1.266 × 10^−5^), indicating a singular fit. To assess overdispersion, we fitted a full OLRE model that included random intercepts for both tank and a unique cage-level observation identifier (obsID). We also fitted a simplified OLRE model that included only the observation-level random intercept. In the full model, the tank and observation-level variances were 2.241 × 10^−13^ and 2.893 × 10^−10^, respectively, and were both close to zero. The full model had an AIC of 66.22, compared to 64.22 for the original model. In the simplified model, the observation-level variance was 2.235 × 10^−10^ and the AIC remained 64.22. The beta-binomial model did not converge and produced an extremely large dispersion estimate of 7.1 × 10^8^; therefore, it was not used for inference. The near-zero OLRE variance estimates and unchanged AIC provided no evidence of overdispersion in the converged models. The binomial GLMM showed a significant effect of water exchange rate on survival [likelihood-ratio test: χ^2^(2) = 16.58, *p* < 0.001]. Survival was lower in the low water exchange group than in the high water exchange group [odds ratio (OR) = 0.09, 95% confidence interval (CI) = (0.03, 0.25), *p* < 0.0001]. It was also lower than that in the medium water exchange group [OR = 0.22, 95% CI = (0.11, 0.46), *p* < 0.0001]. The medium and high water exchange groups did not differ significantly [OR = 0.39, 95% CI = (0.12, 1.26), *p* = 0.117]. Treatment effect estimates and pairwise conclusions were unchanged across the original and converged OLRE models, indicating that the singular fit did not alter the conclusion of lower survival under low water exchange conditions.

**Figure 2:**
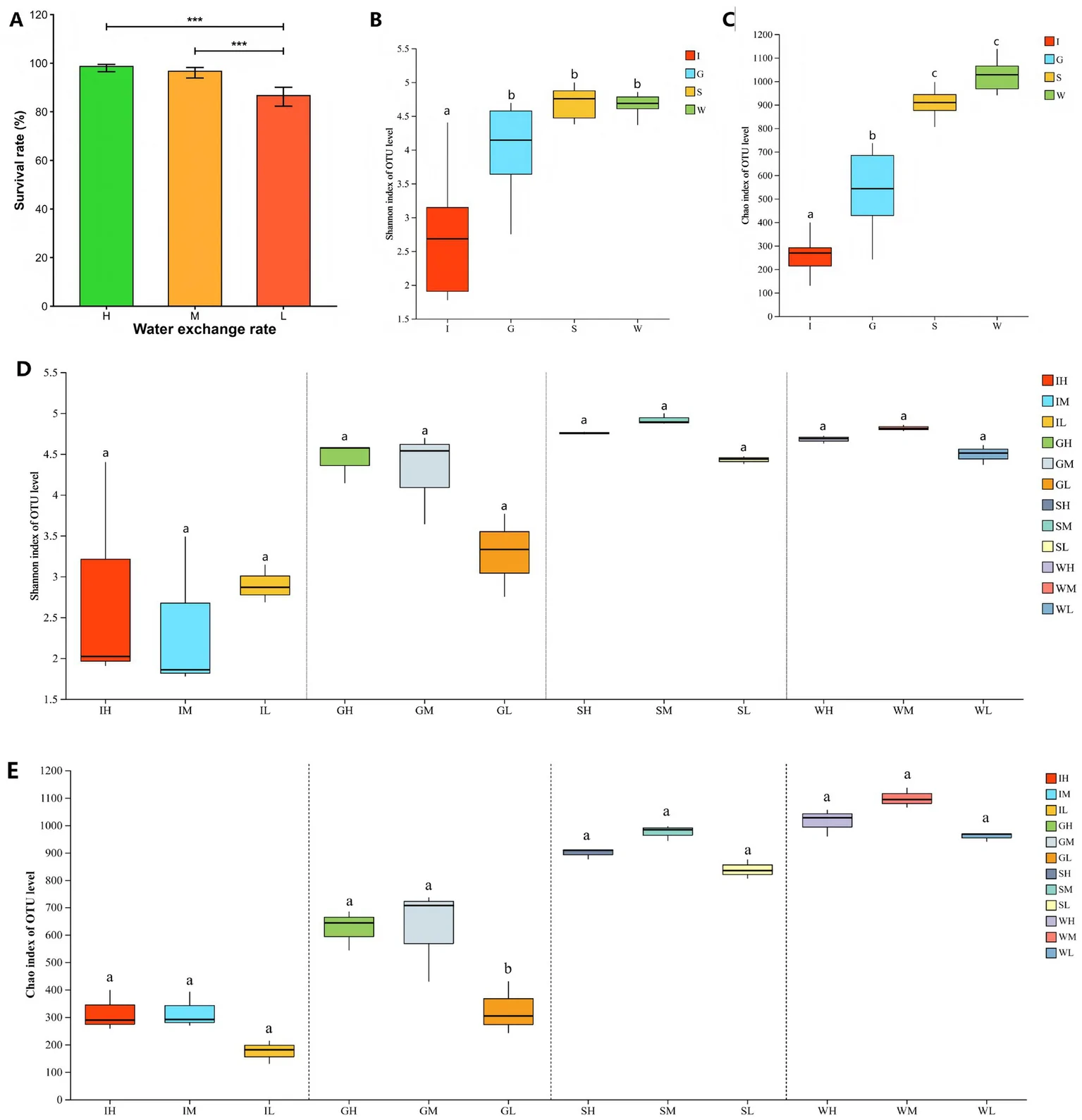
Microbial α-diversity indices (Shannon and Chao1 indices). Survival rates **(A)** of ivory shells treated under different water exchange conditions. Shannon **(B)** and Chao1 **(C)** indices for intestinal, gill, sand, and water samples from ivory shells. Shannon **(D)** and Chao1 **(E)** indices for intestinal, gill, sand, and water samples from ivory shells under different water exchange conditions. IH, IM, IL, GH, GM, GL, SH, SM, SL, WH, WM, and WL represent intestinal, gill, sand, and water samples from ivory shells under high, medium, and low water exchange conditions, respectively.

To investigate the effects of varying water exchange rates on the microbial composition and structure in the intestines, gills, and culture environment (sand and water) of ivory shells, 36 samples were collected from the intestine (I), gills (G), sand (S), and water (W) under high (H), medium (M), and low (L) water exchange conditions. These samples were then sequenced using high-throughput sequencing. Post-quality control, 683,388 sequence reads were obtained, resulting in 23,266 OTUs at 97% similarity. The numbers of OTUs in the intestine, gill, sand, and water samples were 2093, 4,288, 7,884, and 8,510, respectively. The Shannon and Chao1 rarefaction curves indicated adequate sequencing depth (Supplementary Figure S1).

The Shannon and Chao1 indices effectively quantify the diversity and richness of microbial communities. We employed the Kruskal–Wallis *H* test to analyze these indices for the intestine, gills, and surrounding microbial communities of ivory shells under varying water exchange conditions (Figures 2B,C). The findings revealed that environmental microorganisms (sand and water) exhibited greater diversity (Shannon index) and abundance (Chao1 index) compared to host microorganisms (intestine and gills). The gut microbiota showed the lowest indices, significantly differing from other samples, including gill samples (Shannon index: *p* = 0.003; Chao 1 index: *p* = 0.002), sand samples (Shannon index: *p* = 4.9 × 10^−7^; Chao1 index: *p* = 3.7 × 10^−12^) and water samples (Shannon index: *p* = 4.9 × 10^−7^; Chao1 index: *p* = 6.7 × 10^−13^). Although the Chao1 index for the gills differed significantly from that for the surrounding environment (sand: *p* = 2.9 × 10^−7^; water: *p* = 9.2 × 10^−10^), no significant differences were observed in the Shannon index. Further analysis of α-diversity under different water exchange conditions (Figures 2D,E) indicated no significant differences in the Shannon index among the intestinal, gill, sand, and water microbial communities. However, the Chao1 index of the gills at low water exchange rates was notably lower compared to medium (*p* = 0.025) and high (*p* = 0.025) water exchange rates. No statistically significant difference was detected in the α-diversity of the gut microbiota across water exchange rates, whereas low water exchange rates significantly reduced the richness of gill microbial communities.

### 3.2 Microbial community structure

By annotating bacterial taxonomic information and comparing the abundance of corresponding bacterial groups across samples, we identified the top 10 phyla and 20 genera with the highest mean relative abundance to compare the structural composition of the intestine, gills, and surrounding microbial communities of ivory shells (Figures 3A,B). Pseudomonadota was the most abundant phylum, accounting for 69.7%, 64.6%, 52.9%, and 47.4% of the microbiota in the intestine, gills, water, and sand, respectively, indicating it was the most abundant phylum in both host-associated and environmental microorganisms. Bacteroidota was the second most abundant phylum, with higher relative abundance in the environment (26.8% in sand and 20.1% in water) than in the intestine (5.4%) and gills (11.7%). Bacillota had the highest relative abundance in the intestine (19.4%), followed by the gills (9.0%), and the lowest in the environment (0.5% in sand and 1.4% in water). The five most abundant genera across the host and environmental microorganisms were *Vibrio*, *Acinetobacter*, *Ruegeria*, *Nautella,* and *Mycoplasma*. The relative abundance of *Vibrio*, *Nautella*, and *Mycoplasma* was much higher in the intestine (17.6%, 18.1%, and 16.1%, respectively) than in the gills and environment, with significant differences (Kruskal–Wallis *H* test, *p* < 0.05). *Acinetobacter* and *Cohaesibacter* were primarily found in the gills (16.1%, 5.5%), followed by the intestine (6.9%, 2.3%), and were almost absent in the environmental samples (Water: 0.02%, 0.05%; Sand: 0.09%, 0.05%). LEfSe analysis (LDA > 4.0) was used to examine microbial structural composition across the intestine, gills, sand, and water samples (Figure 4A). Bacillota and Pseudomonadota were identified as phylum-level biomarkers of the intestinal microbiota, Bacteroidota as a biomarker of the sand microbiota, and Actinomycetota and Patescibacteria as biomarkers of the water microbiota. At the genus level, *Nautella* and *Vibrio* were identified as biomarkers of the intestinal microbiota, *Acinetobacter* and *Cohaesibacter* as biomarkers of the gill microbiota, Ruegeria as a biomarker of the sand microbiota, and *g_norank_f_Marinifilaceae* and *g_norank_f_Paludibacteriaceae* as biomarkers of the water microbiota. These findings highlight significant differences between the microbial communities in the intestine and gills of ivory shells compared to their surrounding environment.

**Figure 3:**
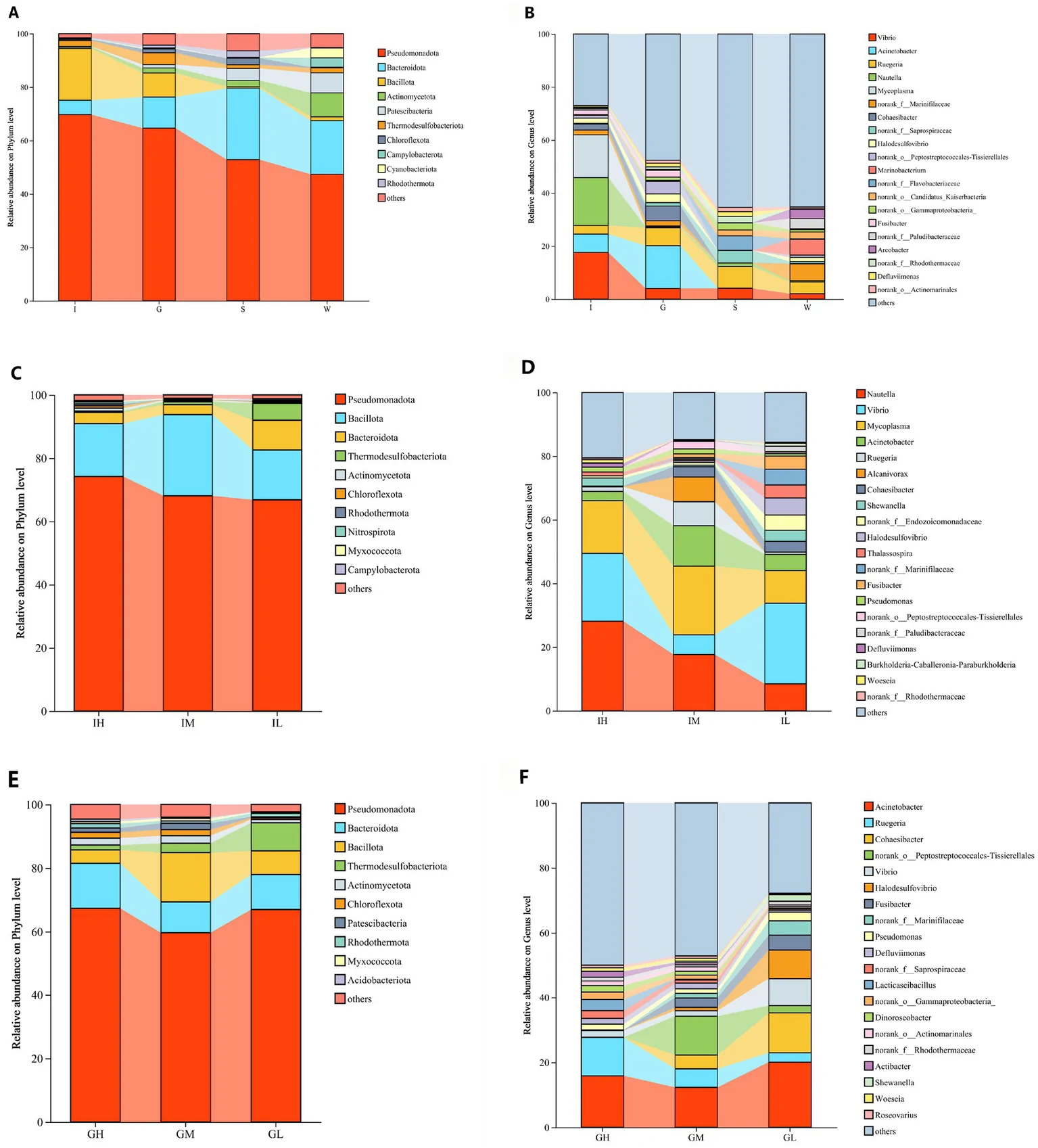
Stacked bar charts showing microbial species distribution (top 10 phyla and top 20 genera). Stacked bar charts showing intestinal, gill, sand, and water microbial species distribution at the phylum and genus levels **(A,B)**. Stacked bar charts showing gut microbial species distribution at the phylum and genus levels **(C,D)** under different water exchange conditions. Stacked bar charts showing gill microbial species distribution at the phylum and genus levels **(E,F)** under different water exchange conditions. Values are presented as the mean of the replicate samples.

**Figure 4:**
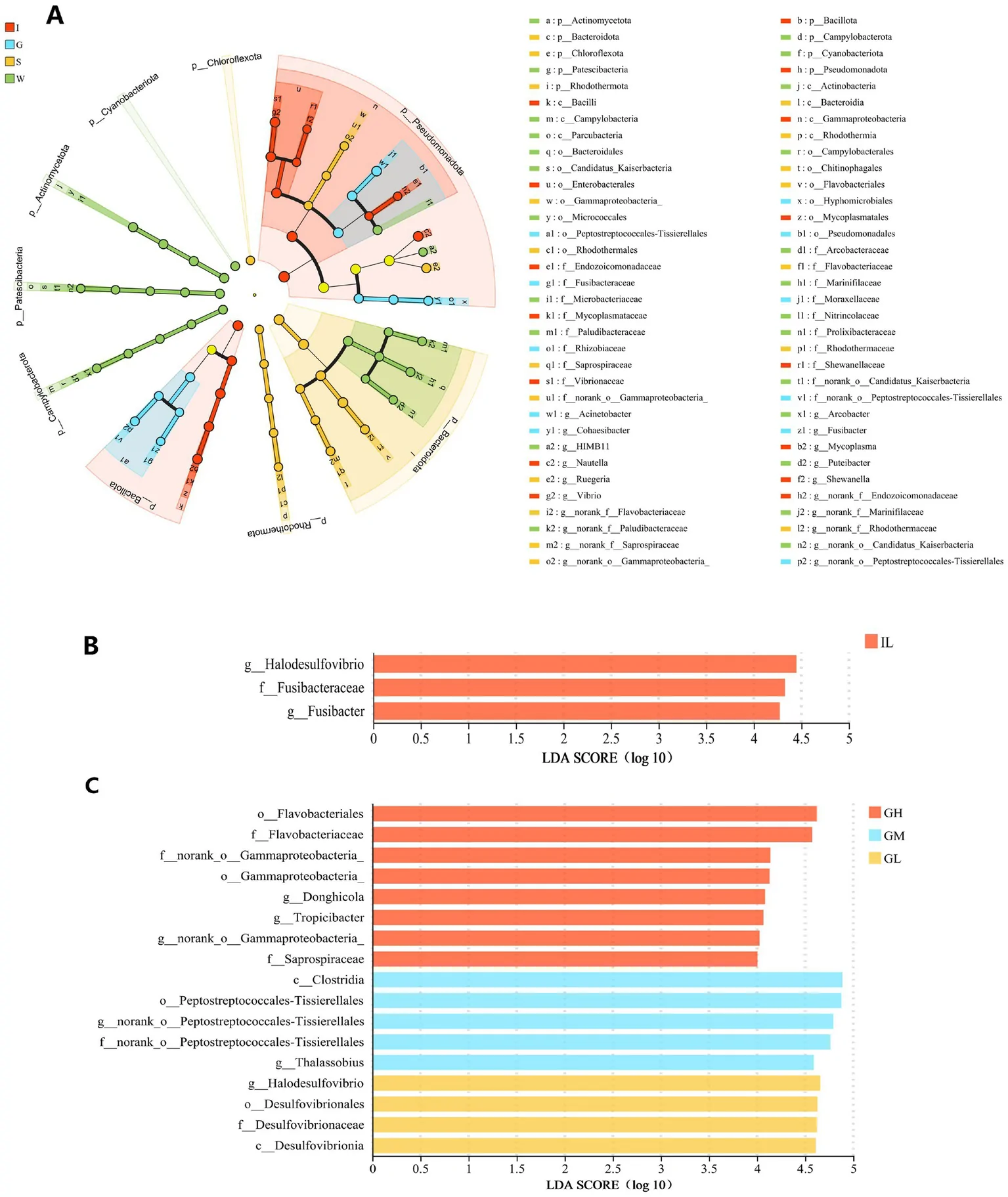
LEfSe analysis revealed community differences among microbiome samples (LDA > 4.0). **(A)** Cladogram showing differentially abundant taxa across multiple taxonomic levels among the intestine (I, *n* = 9), gill (G, *n* = 9), sand (S, *n* = 9), and water (W, *n* = 9) microbiomes of the ivory shell. Nodes colored according to group indicate taxa that were significantly enriched and contributed to inter-group differences. **(B)** LDA score bar plot showing the intestinal microbiota in the ivory shell at high (IH, *n* = 3), medium (IM, *n* = 3), and low (IL, *n* = 3) water exchange rates, with significant enrichment detected only in the low water exchange rate group (IL). **(C)** LDA score bar plot showing the gill microbiota in the ivory shell at high (GH, *n* = 3), medium (GM, *n* = 3), and low (GL, *n* = 3) water exchange rates.

We quantitatively compared the mean relative abundance of the gut and gill microbiota in ivory shells at varying water exchange rates, using proportional stacked bar charts to display the findings. At the phylum level, Pseudomonadota, Bacillota, and Bacteroidota were predominant in both the intestine and gills (Figures 3C,E). At the genus level, *Nautella* (18.1%), *Vibrio* (17.6%), *Mycoplasma* (16.1%), and *Acinetobacter* (6.9%) were the dominant genera in the intestine. The mean relative abundance of *Nautella* decreased with decreasing water exchange rates (28.1%, 17.7%, and 8.4% for high, medium, and low rates, respectively) (Figure 3D). In the gills, *Acinetobacter* (16.1%) and *Cohaesibacter* (5.5%) were the dominant genera, with *Acinetobacter*’s abundance initially decreasing and then increasing as water exchange rates decreased. The mean relative abundance of *Ruegeria* in the gills decreased with decreasing water exchange rates (11.8%, 5.7%, and 2.9% for high, medium, and low rates, respectively), while *Cohaesibacter*, *Vibrio*, and *Halodesulfovibrio* showed increasing trends with decreasing water exchange rates (Figure 3F). LEfSe analysis further revealed distinct microbial community responses to different water exchange rates (LDA > 4.0) (Figures 4B,C). *Donghicola* and *Tropicibacter* were identified as gill-specific biomarkers at high water exchange rates, while *Thalassobius* was identified as a biomarker of the gill community at medium water exchange rates. *Fusibacter* was identified as a biomarker of the intestinal community, while *Halodesulfovibrio* was identified as a shared biomarker of both the intestinal and gill communities at low water exchange rates. These findings describe variation in the relative abundance of specific intestinal and gill genera across water exchange conditions.

### 3.3 Correlation of microbial communities

We performed a quantitative analysis of unique and shared OTUs among the intestinal, gill, and environmental microorganisms of ivory shells. The UpSet plot (Supplementary Figure S2) shows that the intestinal microbiota contained 746 OTUs, of which four were unique and 31 OTUs were shared with the environment. The gill microbiota contained 1,140 OTUs, including eight unique OTUs and 337 OTUs shared with the environment, which indicates that the number of OTUs shared between the gill microbiota and the environment was higher than that between the intestinal microbiota and the environment. The Venn diagrams (Figures 5A–C) show that, as the water exchange rate decreased, the number of unique OTUs in the host (intestine and gills) declined (114, 114, and 103 in the H, M, and L groups, respectively), while the number of unique OTUs in the environment (sand and water) increased continuously (566, 608, and 886 in the H, M, and L groups, respectively). The number of OTUs shared between host and environmental microorganisms was lower under low water exchange conditions (498 OTUs) than under medium (818 OTUs) and high (805 OTUs) water exchange conditions.

**Figure 5:**
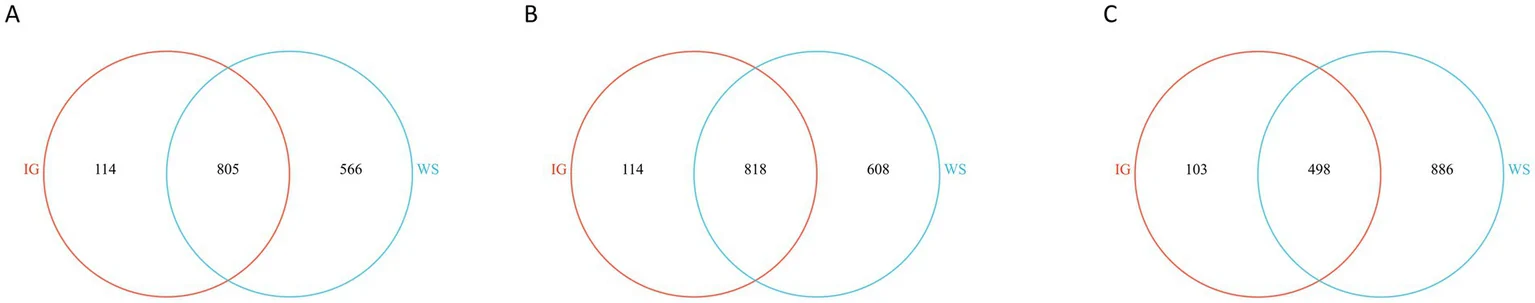
Unique and shared OTUs between host (the intestine and gills) and environment (sand and water) microorganisms under high **(A)**, medium **(B)**, and low **(C)** water exchange conditions.

Using PCoA based on the Bray–Curtis dissimilarity, we examined bacterial composition differences in the intestine, gills, sand, and water at varying water exchange rates. The PC_1_ and PC_2_ axes accounted for 28.1 and 18.6% of the total variation, respectively (Figure 6A). The bacterial community structures of the intestine, gill, sand, and water samples were clearly clustered, indicating significant differences in microbial community composition (R = 0.739, *p* = 0.001). Water samples were positioned on the upper side of the PC2 axis, while intestine, gills, and sand samples appeared on the lower side, suggesting greater similarity between host microorganisms and the sand microbial community. To further assess the contributions of different environmental samples to the host-associated microbial community, we employed SourceTracker analysis, a Bayesian-based approach, to investigate the relative contributions of the surrounding environment at varying water exchange rates. The environmental samples (sand and water) were designated as the “source,” and the host samples (intestine and gills) were considered the “sink” (Figure 6B). Pooled means across water exchange rates and treatment-specific SourceTracker estimates are provided in Supplementary Table S1. The right side of Figure 6B illustrates the contribution of sand and water bacterial communities to the intestine and gills. Approximately 36.8 and 48.2% of the intestine and gill microorganisms were derived from sand, 23.4 and 21.4% were derived from water, and 39.8 and 30.4% were derived from unknown sources (representing microbial communities that could not be assigned to any known source by the Bayesian model). This suggests that sand is the primary source of microorganisms for the intestine and gills of ivory shells. The left side of Figure 6B shows the contribution of sand and water bacterial communities to the intestinal and gill microbiomes at different water exchange rates. As the water exchange rate decreased, the contribution of the sand bacterial community to the intestinal and gill microbiomes declined progressively. Interestingly, compared to the high water exchange rate group, the contribution of the water bacterial community to the intestinal and gill microbiomes increased significantly in the low water exchange rate group, with increases of 20.2 and 28.7%, respectively. In the low water exchange rate group, the contribution of water microorganisms to the intestinal microbiome exceeded that of sand microorganisms, and the contribution to the gill microbiome was equivalent to that of sand microorganisms. These results describe variation in the estimated environmental source contributions to host-associated microbiota across water exchange conditions.

**Figure 6:**
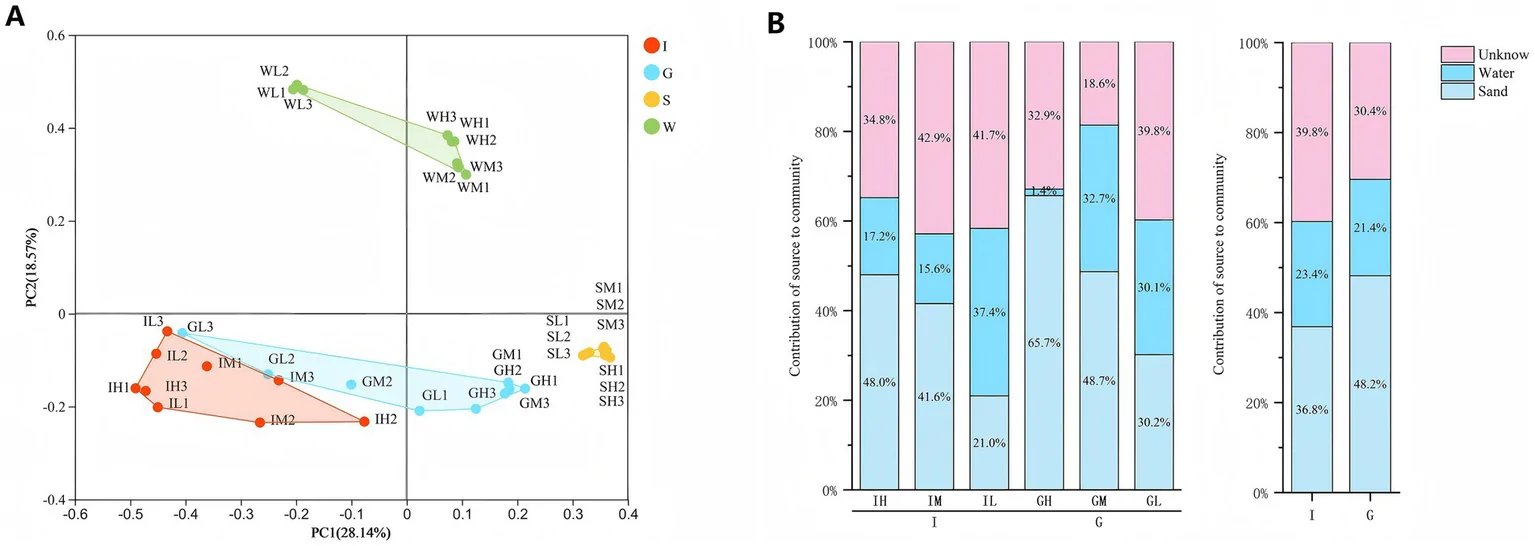
PCoA based on the Bray–Curtis distance matrix and SourceTracker analysis. PCoA of the intestinal, gill, sand, and water samples at different water exchange rates **(A)**. SourceTracker analysis of the contribution of water and sand to the intestinal and gill microbiota at different water exchange rates **(B)**.

### 3.4 Correlation between microbial communities and environmental factors

We employed RDA to explore the relationship between environmental microorganisms and environmental factors, treating microbial community data as the response variable and aquaculture environmental physicochemical properties (Variance Inflation Factor, VIF ≤ 10) as explanatory variables. The water quality variables included in the RDA are presented in Supplementary Table S2. Figure 7 presents the RDA results. RDA plots indicate that RDA_1_ and RDA_2_ explained 40.4 and 16.8% of the variance, respectively. Nitrate (NO_3_^−^-N) was the most significant environmental factor influencing the microbial community (envfit analysis, *r^2^* = 0.75, *p* = 0.001), followed by pH (envfit analysis, *r^2^* = 0.56, *p* = 0.006). The microbial communities in sand clustered in the first quadrant of the RDA ordination, showing a positive correlation with NH_4_^+^-N and NO_2_^−^-N and a negative correlation with pH. Conversely, water microbial communities under high and medium water exchange conditions were grouped in the third quadrant, exhibiting positive correlations with pH and negative correlations with NH_4_^+^-N and NO_2_^−^-N. Only those in the low water exchange treatment clustered in the second quadrant, showing a positive correlation with NO_3_^−^-N.

**Figure 7:**
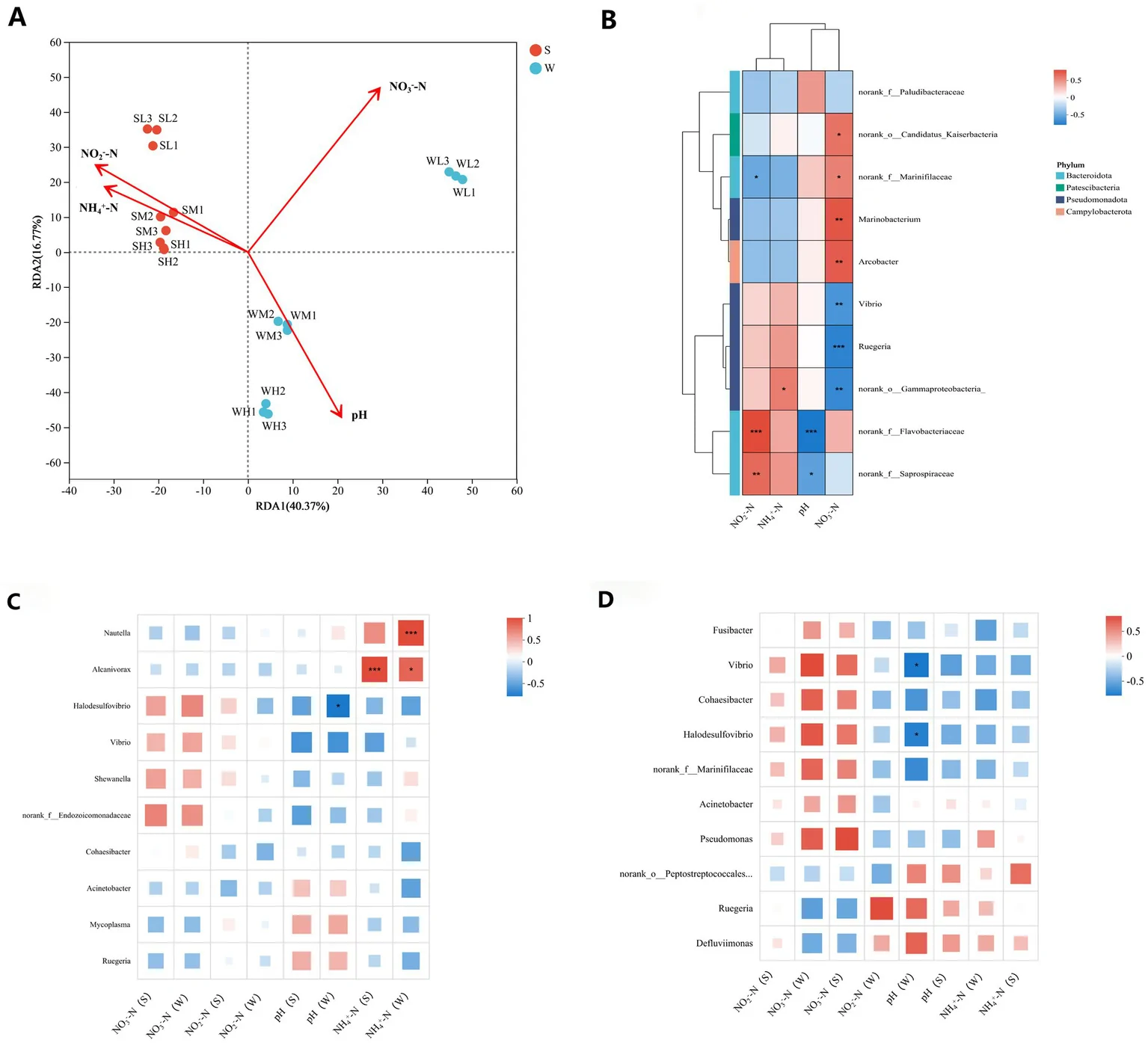
RDA and heatmap analysis of the relationship between microbial communities and environmental factors. RDA of microbial communities and environmental factors in sand and water under different water exchange conditions **(A)**; heatmap analysis of the relationships between environmental **(B)**, intestine **(C)**, and gill **(D)** microbial communities and environmental factors in sand and water.

To investigate the link between microbial communities and environmental factors, we selected the top 10 genera of environmental and host microorganisms based on relative abundance and conducted Pearson correlation analysis with environmental factors (Figures 7B–D). For environmental microorganisms (Figure 7B), *Ruegeria* (biomarker of sand) and *Vibrio* (biomarker of intestine) showed significant negative correlations with NO_3_^−^-N, while *g_norank_f_Marinifilaceae* (biomarker of water) was significantly negatively correlated with both NO_3_^−^-N and NO_2_^−^-N. For host microorganisms (Figures 7C,D), the intestinal biomarker *Nautella* was significantly positively correlated with NH_4_^+^-N in aquaculture water. *Vibrio* in the gills was significantly negatively correlated with aquaculture water pH, and *Halodesulfovibrio*, a common genus-level biomarker of both the intestine and gills under low water exchange conditions, was significantly negatively correlated with water pH. These findings, together with SourceTracker analysis results, highlight the importance of managing the culture environment in aquaculture systems. Implementing scientific measures for water and sand quality management can regulate the abundance of key microorganisms, including pathogens, thus providing a healthy and stable environment for the survival of ivory shells.

### 3.5 BugBase-predicted microbial phenotypes

BugBase analysis predicted nine phenotypes in the host-associated and environmental microbial communities. These included “Gram Positive,” “Gram Negative,” “Biofilm Forming,” “Pathogenic,” “Mobile Element Containing,” “Aerobic,” “Anaerobic,” “Facultatively Anaerobic,” and “Oxidative Stress Tolerance” (Figure 8A). The Kruskal–Wallis *H* test (*p* < 0.05) showed that the “Biofilm Forming” phenotype was significantly less abundant in water samples than in host and sand samples, whereas the “Aerobic,” “Facultatively Anaerobic,” “Anaerobic,” and “Gram Positive” phenotypes were significantly enriched in aquaculture water. We further compared the BugBase-predicted putative “Pathogenic” phenotype among intestinal, gill, sand, and water samples using stacked bar plots showing the five most abundant phyla and the 10 most abundant genera (Figures 8B,C). Pseudomonadota and Bacteroidota were the dominant phyla assigned to this putative phenotype, while *Ruegeria*, *Acinetobacter*, *Vibrio,* and *g_norank_f_Marinobacteria* were the major contributing taxa within the ivory shell aquaculture system.

**Figure 8:**
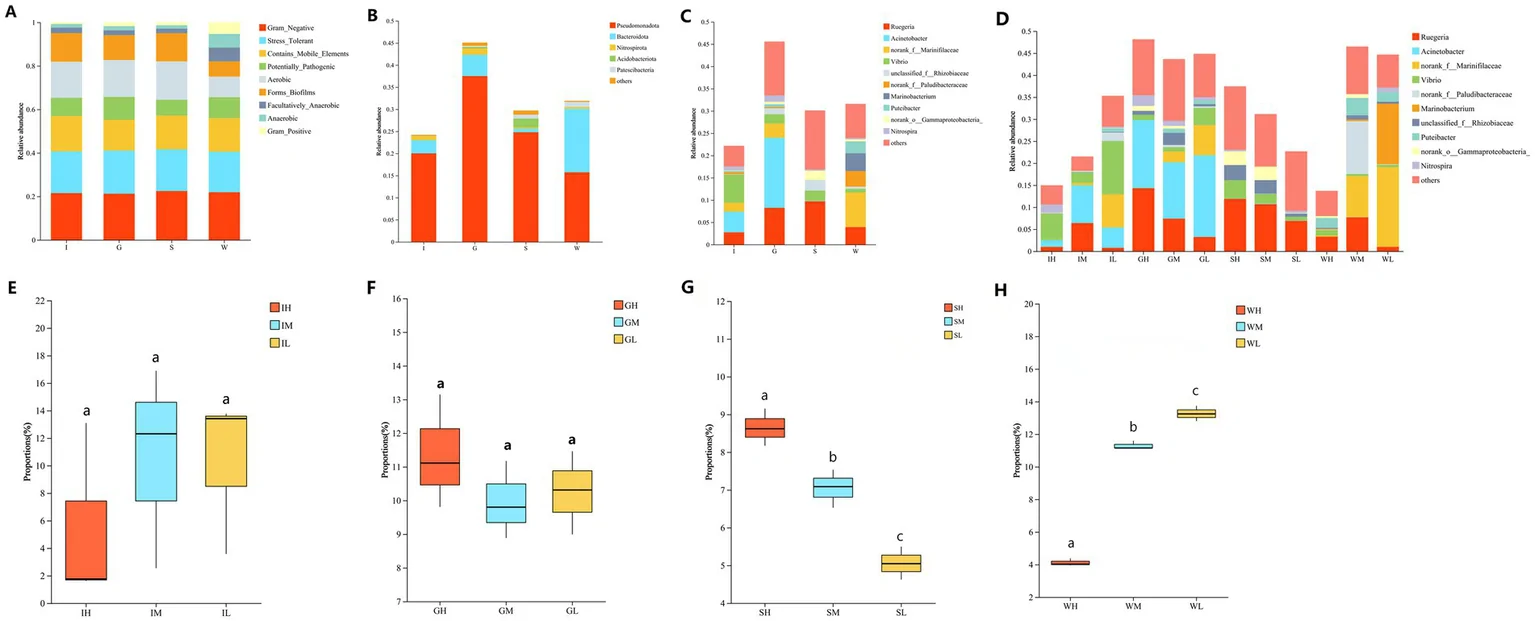
BugBase analysis was performed to evaluate the distribution of predicted bacterial phenotypes and the taxonomic composition of taxa assigned to the putative “Pathogenic” phenotype. Stacked bar plots were used to visualize the comparison of nine putative phenotypes (BugBase-predicted) **(A)** and the putative pathogenic phenotype (BugBase-predicted) in the intestinal, gill, sand, and water microbial communities **(B,C)**. The contributions made by bacterial communities in the intestine, gills, sand, and water to the putative pathogenic phenotype (BugBase-predicted) were analyzed under different water exchange conditions **(D)**. Kruskal–Wallis H test of the relative abundance of putative pathogenic bacteria (BugBase-predicted) in the intestine **(E)**, gills **(F)**, sand **(G)**, and water **(H)** under different water exchange conditions.

To assess the impact of water exchange on the relative abundance of BugBase-predicted putative pathogenic taxa in hosts and environments, we analyzed their contributions to the intestines, gills, sand, and water of the ivory shell aquaculture system (Figure 8D). In the intestine, the relative abundance of putative pathogenic taxa showed an increasing trend as the water exchange rate decreased. Specifically, the highest relative abundance was observed under low water exchange conditions, and *Vibrio* was the predominant contributing genus. However, the Kruskal–Wallis *H* test revealed no statistically significant differences among the three water exchange rates (*p* > 0.05; Figure 8E). In the gills, the relative abundance of putative pathogenic taxa remained high across water exchange rates. The primary contributing genus-level taxa were *Acinetobacter*, *g_norank_f_Marinifilaceae*, and *Vibrio*, accounting for 18.6%, 6.8%, and 3.9%, respectively, with no significant differences observed across water exchange rates (Kruskal–Wallis *H* test, *p* > 0.05; Figure 8F). Conversely, in environmental samples, water exchange significantly altered the relative abundance of BugBase-predicted putative pathogenic taxa in sand and water (Kruskal–Wallis *H* test, *p* = 0.027; Figures 8G,H). Under high water exchange conditions, their relative abundance was highest in sand and lowest in water. In contrast, under low water exchange conditions, their relative abundance in water surpassed that in sand and was highest in the aquaculture environment. The primary contributing genus-level taxa were *g_norank_f_Marinifilaceae* and *g_norank_f_Marinobacteria*, together accounting for 31.9% of the BugBase-predicted putative pathogenic taxa in water. These assignments represent putative phenotype predictions and do not provide direct evidence of virulence or pathogenicity.

## 4 Discussion

The relationship between cultured animals and their environment is closely linked, with environmental microbiota significantly shaping the assembly and adaptation of host gut and gill communities, which directly impacts their adaptability and growth performance (Chen et al., 2022). Therefore, understanding the relationships among intestinal microbiota, gill microbiota, and the aquaculture environment is essential for clarifying how host-associated microbial communities adapt to culture conditions and for managing the health of cultured organisms through environmental regulation (Sun et al., 2019). In this study, we found that the intestine and gills of ivory shells harbored distinct microbial community structures and that host-associated microbiota were associated with environmental microbial communities and water quality variables at different water exchange rates.

### 4.1 Host-associated microbial composition in the intestine and gills

At the phylum level, Pseudomonadota, Bacillota, and Bacteroidota were the dominant phyla in the intestine and gills of ivory shells. The relative abundance of these three phyla was more than 80.0%, which is similar to the findings of previous studies of ivory shells and other aquaculture animals (Zhu et al., 2024; Nic-Matos et al., 2025; Zhao et al., 2025). Pseudomonadota is associated with diverse metabolic processes, and its relative abundance may serve as an indicator of the health status of aquaculture species (Sun et al., 2019). 16S rRNA-based taxonomic characterization revealed that various opportunistic pathogens such as *Vibrio*, *Acinetobacter*, and *Nautella* belong to Pseudomonadota but do not necessarily carry virulence or pathogenic genes. Bacillota can ferment organic compounds such as amino acids and peptides, producing various organic acids (Liang et al., 2026). Bacteroidota is involved in the transformation of organic compounds such as lipids and proteins, decomposing carbohydrates and generating short-chain fatty acids as by-products through fermentation, thereby enabling the host to obtain energy (Corrigan et al., 2015).

At the genus level, the intestine and gills exhibited tissue-specific microbial profiles. *Vibrio*, *Nautella,* and *Mycoplasma* were dominant genera in the intestine of ivory shells. Some members of *Vibrio* and *Mycoplasma* are considered opportunistic pathogens capable of infecting humans and other animals under specific conditions (Destoumieux-Garzón et al., 2020; Li et al., 2017). *Nautella*, however, is regarded as a dominant host-associated genus of uncertain function. The occurrence of opportunistic pathogenic taxa has also been observed in the intestines of 13 aquacultured animals in ponds (Nelson et al., 2012) and in *Apostichopus japonicus* in marine pastures (Cui et al., 2025). Studies indicate that, under appropriate environmental conditions, these pathogens remain benign members of the host microbiome, potentially stimulating the development of the host immune system and maintaining normal immune function (Zhao et al., 2025; Zheng et al., 2017). In contrast, the gill microbial community exhibited a distinct ecological structure, primarily comprising aerobic microorganisms from environmental sources, such as *Acinetobacter*, *Ruegeria*, and *Cohaesibacter*. *Acinetobacter*, known as polyphosphate-accumulating organisms, is extensively used in biological phosphorus removal in sewage treatment plants. This genus can cause various infections, including pneumonia, urinary tract infections, and septicemia, and is known for its broad antibacterial resistance (Zhou et al., 2019). Members of the Roseobacter clade, such as *Ruegeria*, can reduce ammonia nitrogen, nitrate, and hydrogen sulfide concentrations (Brinkhoff et al., 2008; Lenk et al., 2012; Voget et al., 2015), thus enhancing water quality in the gills.

### 4.2 Environmental sources and water exchange-dependent contributions

The microbial composition of the aquaculture environment is a key determinant that shapes the host microbiome and affects its health. Aquaculture animals can selectively recruit microorganisms from the surrounding environment to colonize specific tissues and fulfill their functional roles (Kim et al., 2021). In this study, PCoA revealed that the intestinal and gill microbial communities of ivory shells were more similar to those in the sand samples than those in the water samples, indicating a stronger association between the host and sand-derived microbiota. SourceTracker analysis further supported this finding, with the sand microbial community contributing a larger proportion to the gut and gill microbiomes of ivory shell compared to the water microbiota. This aligns with previous research on other aquaculture species, such as *Penaeus japonicus* (Zhou et al., 2021), *Apostichopus japonicus* (Cui et al., 2025), and *Litopenaeus vannamei* (Huang et al., 2018). However, contrasting findings have been reported for *Micropterus salmoides* (Yang et al., 2024) and *Alligator sinensis* (Wang et al., 2024), where the gut microbiota was more influenced by water than by sediment. These differences in the contributions of environmental microorganisms to host microbiomes may be related to host species and behavioral patterns. Ivory shells exhibit a circadian rhythm, with mollusks lurking in the sand during the day and exposing their siphons, only emerging at night to forage for food (Zou et al., 2024). Similarly, shrimp inhabit the interface between water and sediment, feeding on sediment, which explains the greater influence of sediment on their microbiomes.

The water exchange rate further altered the estimated source composition of the intestinal and gill microbiota. Under high water exchange conditions, the microbiota in the intestine and gills were predominantly derived from the sand substrate. However, as the water exchange rate decreased, the contribution of sand-derived microbes declined progressively. At the low water exchange rate, the intestinal microbiome was primarily sourced from water (21.0% from sand and 37.4% from water), while the gill microbiome showed similar contributions from sand-derived (30.2%) and water-derived (30.1%) microorganisms. These findings suggest that the water exchange rate may alter the source and composition of the intestinal and gill microbiomes in this organism. Notably, these source-tracing results provide only preliminary mechanistic clues about connections between host microorganisms and the environment. The specific functional changes in microbes driven by the water exchange rate, and their reproducibility in real aquaculture systems, still require in-depth validation. Based on the observed microbial source patterns, a preliminary probiotic-based regulation strategy can be proposed: At high water exchange rates, slow-release probiotic particles could be embedded in the bottom sand to optimize the sand microbial community; at low water exchange rates, such as in recirculating aquaculture systems (RAS), water-soluble probiotics could be added to the water to improve the efficiency of regulating host-associated microbial communities. Nevertheless, their practical efficacy must be confirmed through rigorous on-farm trials.

### 4.3 Water quality variables and exploratory microbial associations

Water quality variables were also associated with the structure of environmental and host-associated microbial communities (Gao et al., 2023). RDA identified NO_3_^−^-N and pH as the primary environmental variables associated with microbial community composition in the ivory shell aquaculture environment. This finding is broadly consistent with Sun et al. (2022), who reported that NO_3_^−^-N and pH were two of the key water quality variables associated with microbial richness and diversity in aquaculture pond water. For host-associated microbiota, pH and NH_4_^+^-N were the main environmental variables showing associations with microbial community variation (Zhang et al., 2016). Previous studies have demonstrated that the growth performance of the ivory shell is significantly compromised when the ammonia concentration in the culture system reaches or exceeds 5.0 mg/L (Zhou et al., 2023). The toxicity of ammonia intensifies with increasing pH and ammonia levels. Across four ammonia concentration gradients (0.02, 1.02, 5.10, and 10.20 mg/L), ivory shells exhibited a higher frequency of back-flopping behavior over time at pH 9.0 than at pH 7.0 (Ding et al., 2024).

Existing evidence also indicates that pH is one of the major factors shaping marine microbial communities (Ding et al., 2024), while inorganic nitrogen levels may be associated with the relative abundance of potential pathogenic taxa such as *Vibrio* (Sun et al., 2019). Changes in inorganic nitrogen concentrations may impair animal immune function and increase susceptibility to disease (Jensen, 2003; Zhou et al., 2023). Under low water exchange conditions, microbial community richness decreased in both the intestine and gills, whereas the relative abundance of *Halodesulfovibrio* increased and showed a negative association with pH in aquaculture water. As a sulfate-reducing genus, *Halodesulfovibrio* participates in the terminal step of the anaerobic food chain by oxidizing organic carbon and reducing sulfate to hydrogen sulfide (H_2_S), which may negatively affect the host intestine and gills (Marquis et al., 2021). However, the genus-level correlations in this study should be interpreted as exploratory associations rather than confirmatory evidence.

### 4.4 BugBase-predicted putative pathogenic phenotypes

BugBase analysis showed that, as the water exchange rate decreased, the relative abundance of taxa assigned to the putative “Pathogenic” phenotype increased in the intestine and water but decreased in sand. Integrating the BugBase predictions with the SourceTracker results, these patterns were consistent with the finding that water contributed more than sand to the intestinal microbiome under low water exchange conditions. However, BugBase infers only coarse genus-level phenotypes from 16S rRNA gene profiles using the now-deprecated Greengenes gg_13_5 database. Consequently, these combined analyses cannot establish that putative pathogenic taxa were transmitted from aquaculture water to the intestine, colonized the host, or possessed virulence traits. In addition, the relative abundance of microorganisms assigned to the BugBase-predicted putative “Pathogenic” phenotype in the gills remained high across different water exchange rates, which may be related to the functional role of the gills as a biological interface. As a key barrier to environmental exposure, the gills play an important role in immune recognition and the screening of microbial species entering internal tissues (Peatman et al., 2015).

In total, three limitations of this study should be acknowledged. First, water exchange treatments were applied at the tank or water system level, and limited tank-level replication constrains treatment-level inference. Pooling intestinal and gill samples ensured sufficient DNA for sequencing but limited assessment of individual-level variation. Second, 16S rRNA sequencing captures taxonomic composition but not microbial function or virulence potential; therefore, BugBase predictions and genus-level correlations should be regarded as exploratory findings. The absence of extraction blanks limits the reliable identification and ecological interpretation of low-abundance taxa in low-biomass host tissues. Third, water quality variables and microbial communities were assessed only at the end of the experiment; therefore, longitudinal water quality and microbiome data were unavailable, and functional metagenomic and host physiological data were not collected. Thus, we cannot determine whether the reduced survival observed under low water exchange resulted from microbiome-mediated effects, direct toxicity from nitrogenous compounds, pH-related stress, or a combination of these factors. Future studies should combine independent tank-level replication with time series monitoring of the microbiome and water quality, shotgun metagenomics, culture-based isolation, and host physiological measurements to clarify these mechanisms.

## 5 Conclusion

This study characterized host-associated and environmental microbial communities in ivory shell aquaculture under three water exchange rates using 16S rRNA gene V3-V4 amplicon sequencing. The intestinal and gill microbiota formed distinct host-associated communities that differed from those of the sand and water microbiota. The water exchange rate was associated with shifts in community composition, discriminatory biomarkers, shared and unique OTUs, and environmental source contributions. Low water exchange was associated with lower survival. As water exchange decreased, estimated sand-derived contributions to the host microbiota and the abundance of intestinal *Nautella* and gill-associated *Ruegeria* also declined. LEfSe identified *Halodesulfovibrio* as a shared biomarker in both tissues under low exchange. RDA identified NO_3_^−^-N and pH as the environmental variables most strongly associated with environmental microbial community structure. Exploratory genus-level correlations further showed a negative association between rearing water pH and *Halodesulfovibrio* in both tissues. Together, these findings suggest that water exchange management may influence host–environment microbial connectivity and inform targeted microecological management in ivory shell aquaculture. Future studies should combine independent tank-level replication with time series monitoring of the microbiome and water quality, shotgun metagenomics, culture-based isolation, and host physiological measurements. These approaches could provide further insight into microbial transmission and how microbiome shifts and direct water quality stress may jointly influence host survival under low water exchange conditions.

## Data Availability

The datasets presented in this study can be found in online repositories. The names of the repository/repositories and accession number(s) can be found in the article/Supplementary material.
