# Supplementary material for: Elucidating the relationships between host-associated (intestinal and gill) and environmental (water and sand) microbial communities across water exchange rates in Babylonia areolata aquaculture
Source: Front Microbiol. 2026 Oct 29;17:1890193. doi: 10.3389/fmicb.2026.1890193 (PMC13754835; doi:10.3389/fmicb.2026.1890193)
Supplement: Supplementary file 1 [file Data_Sheet_1.DOCX]

**Supplementary Materials**

**3 Results**

**3.1 Sequencing depth and α-diversity**

**Supplementary Figure S1** Rarefaction curves of microbial communities across sample types and water-exchange treatments. Rarefaction curves were generated for intestinal, gill, water, and sand samples under high (H, 100% d^-1^), medium (M, 50% d^-1^), and low (L, 0% d^-1^) water-exchange treatments. Curves show the Shannon diversity index (A) and the Chao1 richness estimator (B) at the OTU level as functions of the number of reads sampled. The Shannon curves generally reached stable plateaus, and the Chao1 curves showed clear tendencies toward saturation at higher sequencing depths. IH, IM, IL, GH, GM, GL, SH, SM, SL, WH, WM, WL represent Intestine, Gills, Sand and Water samples of ivory shells under High, Medium and Low water-exchange rates, respectively.

**3.3 Correlation of microbial communities**

We performed a quantitative analysis of unique and shared Operational Taxonomic Units (OTUs) among the intestinal, gill, and environmental microorganisms of ivory shell. These results were visualized using an UpSet plot (Fig. S1). The intestinal microbiota contained 746 OTUs, which were classified into a total of 12 phyla, 18 classes, 40 orders, 55 families and 82 genera. Among these OTUs, 4 were unique to the intestine. Of the remainder, 42 were shared with the gill microbiota, 7 were shared with the sand samples, and 14 were shared with the water samples, accounting for 5.63%, 0.94% and 1.88% of the total intestinal OTUs, respectively. The gill microbiota contained 1140 OTUs, which were classified into a total of 27 phyla, 39 classes, 73 orders, 107 families, and 165 genera. Among these OTUs, 8 were unique to the gills. Of the remainder, 42 were shared with the intestinal microbiota, 60 were shared with the sand samples, and 45 were shared with the water samples, accounting for 3.68%, 5.26%, and 3.95% of the total gill OTUs, respectively. Overall, the number of shared OTUs between the gill microbiota and environmental samples was higher than that between the intestinal microbiota and environmental samples.

**Supplementary Figure S2** Unique and common OTUs in the intestine, gills, sand and water. I, G, S, W represent Intestine, Gills, Sand and Water samples, respectively. The values represent the number of OTUs in each group and the shared OTUs between groups.

**Supplementary Table S1.** SourceTracker-estimated contributions (%) of sand, water, and unknown sources to the intestinal and gill microbiomes of ivory shell. Pooled-across-rate means were calculated as the arithmetic means of the treatment-specific estimates for the high-, medium-, and low-exchange treatments.

| Sink tissue | Source | Pooled-across-rate means (%) | Treatment-specific estimates (%) | | |
| --- | --- | --- | --- | --- | --- |
|  |  |  | High exchange | Medium exchange | Low exchange |
| Intestine | Sand | 36.8 | 48.0 | 41.6 | 21.0 |
| Intestine | Water | 23.4 | 17.2 | 15.6 | 37.4 |
| Intestine | Unknown | 39.8 | 34.8 | 42.9 | 41.7 |
| Gill | Sand | 48.2 | 65.7 | 48.7 | 30.2 |
| Gill | Water | 21.4 | 1.4 | 32.7 | 30.1 |
| Gill | Unknown | 30.4 | 32.9 | 18.6 | 39.8 |

**3.4 Correlation between microbial communities and environmental factors**

**Supplementary Table S2.** Endpoint pH and dissolved inorganic nitrogen concentrations in rearing water and sand porewater under different water-exchange treatments on day 60.

| Sample  type | Water-exchange  treatment | Sample  ID | pH | NO_3_^-^-N  (mg·L^-1^) | NO_2_^-^-N  (mg·L^-1^) | NH_4_^+^-N  (mg·L^-1^) |
| --- | --- | --- | --- | --- | --- | --- |
| Water | High (100% d ^-1^) | WH1 | 8.06 | 2.08 | 0.019 | 0.05 |
| Water | High (100% d ^-1^) | WH2 | 8.00 | 1.83 | 0.017 | 0.02 |
| Water | High (100% d ^-1^) | WH3 | 8.13 | 0.78 | 0.011 | 0.07 |
| Water | Medium (50% d ^-1^) | WM1 | 8.00 | 3.32 | 0.011 | ＜0.01 |
| Water | Medium (50% d ^-1^) | WM2 | 8.14 | 1.24 | 0.014 | ＜0.01 |
| Water | Medium (50% d ^-1^) | WM3 | 8.15 | 1.85 | 0.012 | 0.14 |
| Water | Low (0% d ^-1^) | WL1 | 7.91 | 16.23 | 0.012 | 0.07 |
| Water | Low (0% d ^-1^) | WL2 | 7.88 | 14.93 | 0.009 | 0.01 |
| Water | Low (0% d ^-1^) | WL3 | 7.81 | 13.43 | 0.011 | ＜0.01 |
| Sand | High (100% d ^-1^) | SH1 | 7.64 | 1.09 | 0.030 | 0.19 |
| Sand | High (100% d ^-1^) | SH2 | 7.71 | 1.30 | 0.018 | 0.15 |
| Sand | High (100% d ^-1^) | SH3 | 8.05 | 0.68 | 0.016 | 0.30 |
| Sand | Medium (50% d ^-1^) | SM1 | 7.64 | 3.31 | 0.018 | 0.73 |
| Sand | Medium (50% d ^-1^) | SM2 | 8.02 | 0.57 | 0.012 | 0.28 |
| Sand | Medium (50% d ^-1^) | SM3 | 8.06 | 1.22 | 0.025 | 0.21 |
| Sand | Low (0% d ^-1^) | SL1 | 7.32 | 15.02 | 0.028 | 0.16 |
| Sand | Low (0% d ^-1^) | SL2 | 7.69 | 13.41 | 0.060 | 0.29 |
| Sand | Low (0% d ^-1^) | SL3 | 7.26 | 9.01 | 0.016 | 0.04 |

**Note:** Values represent individual endpoint measurements obtained on day 60 from three cage-level sampling units per sample matrix and treatment. Sample IDs indicate the sample matrix (W, rearing water; S, sand porewater), water-exchange treatment (H, high; M, medium; L, low), and cage number (1-3). Sand porewater was obtained by vacuum filtration of sand samples without the addition of an extractant. NO_3_^⁻^-N, nitrate nitrogen; NO_2_^⁻^-N, nitrite nitrogen; NH_4_^+^-N, ammonia nitrogen. The limits of detection were 0.01 mg L^-1^ for NO_3_^⁻^-N and NH_4_^+^-N and 0.003 mg L^-1^ for NO_2_^⁻^-N. Concentrations below the corresponding detection limit are reported as <0.01 or <0.003 mg L^-1^.
